# Comments on the methodology and completeness of a meta-analysis on the association between social frailty and adverse outcomes

**DOI:** 10.1007/s40520-024-02741-7

**Published:** 2024-04-19

**Authors:** Xiao-Ming Zhang, Zhe Yang

**Affiliations:** 1 Department of Emergency, The People’s Hospital of Baoan Shenzhen, Shenzhen, China; 2 Southern Medical University, Guangzhou, 510515 China

Dear Editor:

The concept of social frailty, a burgeoning subtype of frailty prevalent amongst the elderly, has increasingly captivated the attention of geriatric medical researchers [1]. A plethora of studies has delved into the ramifications of social frailty on this demographic. A recent meta-analysis divulged that the prevalence of social frailty in the elderly is an alarming 22% (95% CI:18% 26%), underscoring the substantial burden it imposes [2]. With keen interest, we perused the publication by Li et al., which investigates the repercussions of social frailty on adverse clinical outcomes, revealing that elderly individuals afflicted with social frailty face heightened risks of disability, mortality, and depressive manifestations [3]. The study is commendable for its pioneering exploration of the linkage between social frailty and negative health outcomes, employing a robust methodology encompassing meta-analysis, assessment of publication bias, and sensitivity analysis. We laud the authors for their commendable effort, yet there are several aspects that merit further discussion.

Primarily, the search methodology and inclusion/exclusion criteria employed in Li’s analysis appear to overlook a pivotal study conducted by Gobbens in 2021 [4], which utilized the Tilburg Frailty Indicator to gauge social frailty in community-dwelling elders. This study indicated that social frailty does not exacerbate mortality risk post-adjustment for age and sex, presenting a Hazard Ratio (HR) of 1.17 (95% CI: 0.97–1.40). This omission suggests that this study warrants incorporation into their systematic review and meta-analysis.

Furthermore, we observe a discrepancy in the original study by Lee [5], which demonstrated that individuals with high levels of social frailty exhibited a significantly increased mortality risk compared to their non-socially frail counterparts, evidenced by an HR of 1.45 (95% CI: 1.07–1.97). However, Li et al. extracted the data HR of 3.14 (95% CI: 1.81–5.46), pertaining to individuals with both high levels of social and physical frailty, thereby conflating the effects. It is essential that the impact of social frailty in isolation be discerned and analyzed. Therefore, Li and their colleague should better to use the data of 1.45 not 3.14 regarding the association between social frailty and mortality in their meta-analysis.

**Fig. 1:**
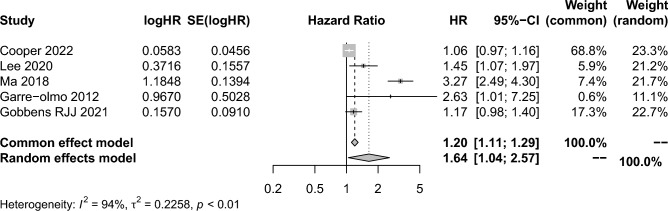
Meta-analysis for the association between social frailty and mortality among community-dwelling older people

Re-evaluation of the original data, considering these factors, yielded a pooled HR for the association between social frailty and all-cause mortality in community-dwelling elders of 1.64 (95% CI: 1.04–2.57), as illustrated in Fig. 1.

Lastly, Li's study posits an inclusion criterion limiting the sample population to community dwellers. However, prior research indicates that social frailty prevalence is significantly higher among hospitalized elderly individuals compared to their community-dwelling counterparts (40% versus 17%, respectively) [2]. This discrepancy prompts an inquiry into whether the impact of social frailty on mortality is more pronounced within the hospitalized demographic. Consequently, we advocate for the execution of a subgroup analysis to elucidate this critical aspect.

In conclusion, while Li and colleagues have significantly contributed to the understanding of social frailty’s impact on mortality among the elderly residing in communities, addressing the aforementioned concerns could substantially enhance the validity and comprehensiveness of their systematic review and meta-analysis.
